# Treatment of Malignant Tracheobronchial Stenosis with Y-Shaped Stent: A Multicenter Retrospective Study

**DOI:** 10.3390/jcm15030966

**Published:** 2026-01-25

**Authors:** Diletta Mongiello, Vincenzo Pagliarulo, Letizia Perri, Domenico Pourmolkara, Francesco Puma, Giovanni Natale, Riccardo Orlandi, Francesco Sollitto, Ugo Cioffi, Angelo Guttadauro, Federico Raveglia, Alfonso Fiorelli, Domenico Loizzi

**Affiliations:** 1 Thoracic Surgery Unit, University of Foggia, 71122 Foggia, Italy; francesco.sollitto@unifg.it (F.S.); domenico.loizzi@unifg.it (D.L.); 2 Cardiothoracic Department, Freeman Hospital, Newcastle NE7 7DN, UK; vincenzo.pagliarulo@nhs.net; 3 Respiratory Diseases Department, University of Modena, 41121 Modena, Italy; letiziaperri9@gmail.com; 4 Thoracic Surgery Unit, University of Perugia, 06100 Perugia, Italy; domenico.pourmolkara@gmail.com (D.P.); francesco.puma@unipg.it (F.P.); 5 Thoracic Surgery Unit, ‘Luigi Vanvitelli’ Hospital, 80131 Napoli, Italy; dott.natale.giovanni@gmail.com (G.N.); alfonso.fiorelli@unicampania.it (A.F.); 6 Thoracic Surgery Unit, Papa Giovanni XXIII Hospital, 24127 Bergamo, Italy; riccardo.orlandi@unimi.it; 7 Department of Surgery, University of Milan, 20122 Milan, Italy; ugo.cioffi@guest.unimi.it; 8 Department of Medicine and Surgery, University of Milano Bicocca, 20158 Milan, Italy; angelo.guttadauro@unimib.it; 9 Department of Thoracic Surgery, Fondazione IRCCS San Gerardo dei Tintori, 20900 Monza, Italy; federico.raveglia@icloud.com

**Keywords:** central airway obstruction, Y-shaped stents, bronchoscopy, malignant airway stenosis

## Abstract

**Objectives:** Central airway obstruction (CAO) caused by malignant tumors may necessitate combined and prompt treatment. The aim is to recanalize and stabilize the airways as palliation. We present our multicentric experience managing malignant CAO through the placement of Y-shaped self-expanding covered metallic or silicone trachea-bronchial stents. **Methods:** This retrospective study includes patients who underwent placement of Y-shaped stents from 2002 to 2024 across six different centers in Italy and Great Britain. We evaluated outcomes related to the feasibility and safety of the procedure, as well as the palliation of dyspnoea on the Modified Borg Scale of Dyspnoea. **Results:** Eighty patients (56.2% female) with a mean age of 64.8 ± 9.6 years were included in the study. Successful placement was achieved in 76 (95%) cases, with no cases of intraoperative mortality. The mean procedure time was 36.64 ± 15.7 min. The complications noted included: 7 (8.7%) cases of periprocedural clinical complications and 7 (8.7%) patients requiring intensive care unit admittance after the procedure. Fifty patients (78.1%) received cancer treatment following the procedure. The mean dyspnoea score on the Borg scale decreased from 7.78 ± 0.98 to 4.02 ± 2.2 (*p* < 0.05). **Conclusions:** The placement of metal or silicone Y-shaped stents is a feasible and safe procedure for the palliative treatment of dyspnoea in patients with malignant stenosis of the trachea and main bronchi. Stabilizing the airway also enables these patients to access cancer treatments.

## 1. Introduction

Central airway obstruction (CAO) is defined as an occlusion involving more than 50% of the trachea or main bronchi [1]. Malignant CAO is usually caused by primary tumors of the lung, esophagus, or thyroid, or by metastases invading the tracheobronchial tree [2]. In some cases, the tumor may involve multiple levels, resulting in complex stenoses [3]. The primary indications for endotracheal or endobronchial stent placement—whether straight or Y-shaped—include airway obstruction due to intrinsic tumor growth or extrinsic compression. Additional indications include malignant tracheoesophageal fistulas (TEF) [4]. Severe tracheobronchial stenosis or fistulae can result in atelectasis, obstructive pneumonia, hemoptysis, or life-threatening respiratory failure. Clinically, patients may start to experience dyspnea when airway patency is reduced by approximately 50% [3]. Symptoms can progress rapidly, and severe obstruction may result in asphyxia, requiring urgent intervention to restore airway patency [5]. When malignant central airway obstruction involves the carinal region, a Y-shaped stent is often required [6]. These stents provide improved stability and reduced migration risk due to their anatomical fit and contact with mucosal surfaces [7]. This study presents a multicenter experience on the use of self-expandable metal and silicone Y-shaped stents for the palliative treatment CAO or TEF. The aim is to evaluate short-term and perioperative outcomes, including feasibility, safety, and technical success of the procedure, intraoperative and perioperative complications, effectiveness in relieving dyspnoea as measured by the Modified Borg Scale (MBS), and postoperative length of stay (LOS). A short follow-up was also conducted to assess early clinical outcomes. A brief narrative review of the literature was also performed to contextualize our findings.

## 2. Materials and Methods

### 2.1. Study Design

This is a retrospective multicenter study enrolling patients who underwent placement of either a Y-shaped silicone or self-expandable metallic stent (SEMS) for CAO between January 2002 and January 2024 at five centers in Italy and one center in the United Kingdom. The study was approved by the Institutional Review Board of the coordinating center, AOU Policlinico of Foggia (protocol code 190/C.E./2024, 14 January 2025). Given the retrospective nature of the study, it was not deemed necessary to request explicit informed consent.

### 2.2. Data Collection

To facilitate data collection, a database was distributed among the centers. The following variables were recorded: demographic information, histology, site of CAO, degree of dyspnoea before and after stent placement, type of bronchoscopy used during the procedure (flexible or rigid), recanalization technique (balloon or laser), fluoroscopy, complications (clinical and technical), procedure duration (in minutes), LOS (in days), and subsequent oncologic treatments. Patient identification data were anonymized by recording only initial letters and were analyzed in an anonymous format.

### 2.3. Statistical Analysis

Descriptive statistics were used for demographic and baseline characteristics. Continuous variables were reported as mean ±standard deviation (SD), and differences assessed by *t*-test. A *p*-value < 0.05 was considered statistically significant. The software STATA (Version 18) was used for statistical analyses.

### 2.4. Indication and Preoperative Setup

Y-shaped stent implantation was indicated in cases of malignant central airway obstruction involving the lower trachea and the main carina, or when one or both mainstem bronchi were compromised. Selection criteria included: (1) severe dyspnoea and risk of death from asphyxia (Borg Scale > 6), (2) stenosis >50% of the airway lumen, (3) possibility of starting or continuing tumor treatment. The choice of stent type depended on the patient’s prognosis, on the stent availability and on the operator’s experience. Silicone stents were preferred in patients with long-term expected survival, or need for potential future stent removal. Before stent placement, all patients underwent chest computed tomography (CT) to confirm the indication and to measure the appropriate stent size and diameter. When clinically feasible, flexible bronchoscopy was performed to assess the cause, location, and severity of the stenosis, as well as to evaluate the need for airway dilatation.

### 2.5. Procedures

The Y stent consists of three components: tracheal, left bronchial, and right bronchial branches. We describe three different techniques for Y-shaped stent placement: with and without fluoroscopic guidance for metallic stents, and the technique for silicone stent deployment.

#### 2.5.1. Metallic Stent Insertion Under X-Ray Guidance

The process starts with the insertion of a rigid bronchoscope into the airway, creating stable access for further intervention. A flexible bronchoscope is then introduced through the rigid bronchoscope to perform a detailed exploration of the tracheobronchial tree and to assess the optimal site for stent placement. The tracheal carina is identified and marked under fluoroscopic guidance externally with a skin marker, used as a reference point for accurate stent positioning. Two guidewires are then advanced: one into the right main bronchus (RMB) and the other into the left main bronchus (LMB), to guide the introducer system. The stent introducer, containing the folded stent, is carefully advanced over the guidewires to the target location. Deployment is performed under fluoroscopic guidance, aligning the stent’s radiopaque carinal marker with the external skin reference to ensure the correct placement within the tracheobronchial anatomy.

#### 2.5.2. Metallic Stent Insertion Without X-Ray Guidance

Under general anesthesia, a rigid metal tracheoscope (12 or 14 mm in diameter, depending on patient size) is introduced through the vocal cords, with or without the aid of a laryngoscope. A complete evaluation of the bronchial tree is then carried out using a flexible bronchoscope, which also allows aspiration of secretions. If necessary, endoscopic recanalization is performed through the rigid bronchoscope using various techniques—mechanical debulking, laser therapy, or balloon dilation—based on the characteristics of the stenosis. Under direct endoscopic visualization, two guidewires are placed: one into the RMB and the other into the LMB. The ideal distal position of the left bronchial branch and the proximal margin of the tracheal branch of the stent are measured from the proximal edge of the tracheoscope using the flexible bronchoscope. These reference distances are then marked directly on the stent introducer with a marker pen. The guidewires are inserted into the respective distal limbs of the preloaded, folded Y-shaped stent. Stent deployment is performed endoscopically by advancing the introducer while keeping the sheath fixed in position.

#### 2.5.3. Silicon Stent Insertion

Tracheal intubation is performed using an 8.5 mm rigid bronchoscope, which is employed to reopen the airway lumen as extensively as possible. Spontaneous assisted ventilation is maintained through the bronchoscope’s channel throughout the procedure. Airway dilation is achieved using different techniques, selected based on the local anatomical and pathological conditions: mechanical tumor debulking with endoscopic forceps, blunt coring with the rigid bronchoscope tip, and, less frequently, laser. The airway must be sufficiently reopened to allow the 8.5 mm bronchoscope to reach the carina and to be advanced into both main bronchi. Haemostasis is achieved through a combination of mechanical tamponade, Nd:YAG laser application, and cold saline irrigation. Before removing the rigid bronchoscope, precise measurements are taken to tailor the stent to the airway segment requiring support. Customization is performed on both tracheal and bronchial limbs of the silicone stent. The proximal and distal ends are trimmed according to the measured lengths and carefully smoothed to reduce the risk of mucosal trauma. Once airway patency and hemostasis are deemed satisfactory and the stent has been customized, the patient is hype oxygenated to minimize the risk of desaturation during the subsequent placement. The stent is grasped with Freitag forceps, ensuring the correct orientation of its bronchial branches. The patient is then extubated, and the rigid bronchoscope is removed. Placement of the silicone Y-stent is carried out under direct visualization using a laryngoscope. In rare cases, the stent can be positioned correctly via the laryngoscope alone. More commonly, the stent is initially released in the distal trachea, and final positioning is achieved through a combined approach using both rigid and flexible bronchoscopes. The procedure should not be concluded unless optimal stent positioning is confirmed.

Following the stent placement procedure, all patients underwent a follow-up chest X-ray.

## 3. Results

### 3.1. Patients and Centers

Between 2002 and 2024, 80 patients underwent Y-shaped tracheal stent placement for CAO at 5 thoracic surgery centers and 1 respiratory disease department, as shown in Table 1.

### 3.2. Study Population

Table 2 presents the baseline characteristics of the study population. The most common underlying malignancy was lung cancer, followed by esophageal cancer. All patients presented with severe dyspnoea at diagnosis, requiring urgent management.

### 3.3. Procedures’ Details

Table 3 summarizes the technical details of the procedures. All procedures were performed under general anesthesia and using a rigid bronchoscope. Recanalization maneuvers prior to stent placement were required in 70 cases. The mean duration of the procedure was 36.7 ± 15.7 min. Successful stent deployment was achieved in 76 patients (95.0%). Unsuccessful was associated with technical complications, occurring in 4 cases (5.0%) of stent release failure requiring removal.

### 3.4. Outcomes

**Figure 1:**
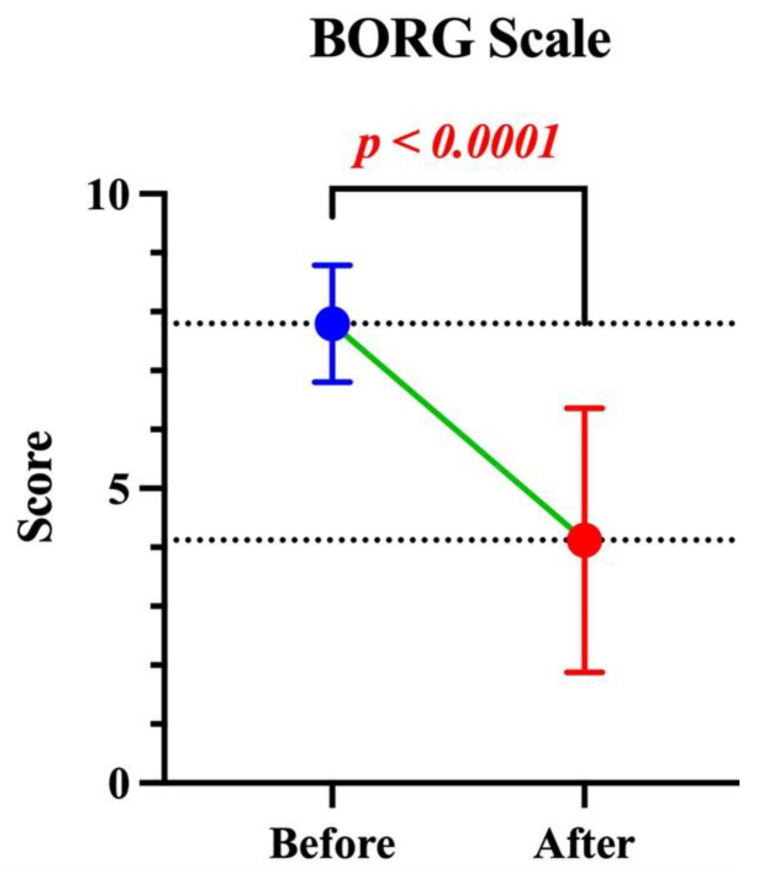
Graph showing the average with a 95% confidence interval of the score given for dyspnoea on the Borg Scale before and after the stent positioning.

Table 4 shows the clinical outcomes. Periprocedural complications occurred in 7 patients (8.7%). Two patients (2.5%) died during the recovery period. The LOS was 3.76 ± 3.1 days. Following airway stabilization with the stent, 50 patients (78.1%) initiated oncologic treatments. The mean dyspnoea score on the MBS decreased significantly from 7.78 ± 0.1 before the procedure to 4.02 ± 2.2 after the procedure (*p* < 0.0001), as illustrated in Figure 1.

## 4. Discussion

The placement of Y-shaped stents for palliative treatment of dyspnoea in patients with CAO for malignancy is safe and effective, with a low incidence of technical and clinical complications. This intervention is currently performed in only a limited number of specialized thoracic surgery or interventional pulmonology centers, with relatively little experience documented in the literature. Table 5 provides a summary of the available studies.

The most common indication for airway stenting is obstruction caused by tumors. Sabath et al. reported that stenting is particularly required in extrinsic or mixed lesions for which pure endoscopic treatment is insufficient to restore adequate airway patency [4]. If tumors involve the carina, the placement of a Y-shaped stent aims to maintain the patency of the trachea, as well as the right and left main bronchi [8]. In the studies summarized in Table 5, central stenosis remains the most frequent indication. Some case series, such as the study by Madan et al., describe patients presenting with both indications simultaneously: in their cohort of 38 patients, 10.5% had both CAO and TEF [9].

Stents are available in metal and silicone, each with distinct advantages and disadvantages. Sabath et al. describe and compare the characteristics of the stent types in a comprehensive review [4]. Silicone stents designed by Dumon are easily inserted and removed, well tolerated by patients, and highly effective in relieving respiratory symptoms [10], but they have a high frequency of migration, and their deployment always requires the rigid bronchoscope.

Wallace et al. first described the use of a metallic stent for bronchial stenosis in 1986 [11]. SEMS are easier to insert, including through a flexible bronchoscope, and have low migration rates, structural stability, and a high degree of dilation. However, when deployed in neoplastic tissue, their removal can be challenging due to tumor ingrowth into the mesh. Additionally, complications such as stent fracture and tracheal ring damage have been reported, likely related to the rigid structure potentially causing mucosal injury. Despite these issues, SEMS remain the most used tracheobronchial stents worldwide [12]. Most authors agree that the choice of stent should be guided by the patient’s expected life expectancy. In terminally patients with severe airway stenosis and significant dyspnoea, a self-expanding metal stent is often preferred due to its ease of placement and durability. Conversely, in cases of benign stenosis or when a neoplastic patient is expected to resume or initiate oncologic treatment following airway restoration, silicone stents may also be appropriate because they are easier to remove, and the tumor’s ingrowth tendency is lower. Both rigid bronchoscopy and general anesthesia provide a safer environment for placing Y-shaped stents in patients with severe dyspnoea and critical CAO. The rigid bronchoscope allows continuous and complete control of the airway throughout the procedure, facilitating accurate stent placement, typically achieved on the first attempt. In the event of partial or displaced stent deployment, it allows prompt intervention to reposition, remove, and immediately restore airway patency. It also facilitates dilation maneuvers prior to prosthesis placement. However, there are reports of airway stenting procedures performed without general anesthesia. Li et al. described a series of 10 patients with CAO treated emergently with metal stents under local anesthesia, reporting no procedure-related complications [8]. Qian et al., in a case series of 102 patients with CAO treated with SEMS reported that 65.7% of the procedures were performed under local anesthesia using a flexible bronchoscope [5]. Stenting requires not only appropriate technical training but also substantial experience in patient selection and complication management. We used the MBS to evaluate dyspnoea, as it correlates well with other clinical parameters and proves useful for assessing and monitoring outcomes in patients experiencing breathlessness. Patients reported a high level of satisfaction with the MBS regarding its ease of use and found that the language adequately captured their experience of dyspnoea [13]. Zeng et al., in their 2022 comparative pilot study on the use of two techniques (through the scope and over the wire) for stent placement in CAO, used the dyspnoea index score to assess dyspnoea outcomes before and after the procedure [14]. According to the study of Mi et al., CT and bronchoscopy remain the most reliable methods for accurately determining the airway dimensions when choosing the stent size to be used [15].

During the procedure, maintaining an adequate airway lumen is essential to ensure effective ventilation. To achieve continuous control throughout the maneuvers, Monnier describes a technique involving laryngoscopy combined with jet ventilation delivered through a 2 mm diameter catheter, which is introduced ahead of the stent delivery system via the laryngoscope. All six procedures performed using this technique were successful, with no episodes of oxygen desaturation reported [16]. Yu Fei Fu [17] also described a method for airway control during stent deployment, involving the use of a catheter connected to an oxygen source, inserted over a guidewire from the nasal cavity to beyond the stenosis and positioned below the carina, under local anesthesia.

Stent deployment is commonly preceded by maneuvers aimed at recanalizing the airway, which can be performed mechanically using a balloon or via laser therapy. The use of a balloon is very useful for rapid dilation and restoration of airway patency in emergencies, but in cases of malignant stenosis, further dilation maneuvers are necessary [18]. Nd:YAG laser therapy has been a well-established treatment modality for CAO since 1980. It has gained recognition as a major palliative intervention due to its minimal side effects and its effectiveness [19].

Comparing our study to other experiences regarding complications, Schulze et al. [7] reported a higher rate of technical complications (29.6%), primarily due to persistent obstruction of the main bronchus or incomplete stent unfolding. Their series also included 6 cases of minor bleeding, 1 case of stent dislocation, and 2 cases of material defects [7]. Sehgal’s study on silicone stents reported stent-related complications in 14 out of 27 cases (52%), 3 procedural complications including dental trauma, enlargement of tracheoesophageal fistulas, and 5 patients requiring endotracheal intubation immediately following the procedure [20].

Several strategies are essential to minimize adverse events associated with Y-shaped airway stents. In our case series, migration risk was reduced by ensuring correct carinal anchoring and selecting stents with adequate branch lengths based on preoperative CT and intraoperative sizing. A chest X-ray performed immediately after the procedure confirmed the correct positioning of the stent and the absence of complications such as pneumothorax, neck emphysema, mediastinal emphysema, airway rupture, or massive bleeding in the pleural cavity. To prevent mucus plugging, all centers adopted a postoperative protocol including humidified oxygen, nebulized saline, and scheduled bronchoscopy when clinically indicated. Infection risk was minimized through perioperative antibiotics and early evaluation of fever or dyspnea. Endoscopic follow-up, also based on patient’s symptoms, enabled early detection of complications, allowing timely interventions.

Being a retrospective study, we conducted only a periprocedural assessment with short-term follow-up, monitoring patients until discharge and for up to three months thereafter. Periprocedural and in-hospital mortality related to the procedure was very low in our series (2.5%) while in the literature it ranges from 0 to 22.6%. Notably, Schulze reported 7 in-hospital deaths among 31 procedures but did not specify the timing of these deaths [7].

An important finding in our experience concerns the feasibility of administering cancer treatments following stent placement. Furukawa et al. demonstrated that airway stenting followed by adjuvant therapy may improve the survival of treatment-naïve patients with severe symptomatic airway obstruction caused by advanced lung cancer [21]. Wang also reported that antitumor therapy, combined with airway stent implantation, significantly prolonged the survival time [22]. As illustrated in Figure 1, the mean dyspnoea score on the BS decreased from 7.78(SD ± 0.1) before the procedure to 4.02(SD ± 2.2) after the procedure. Studies in the literature consistently report symptomatic improvement in most patients following Y-shaped stent placement, with success rates ranging from 75% to 100%. Achieving relief of respiratory symptoms remains the primary goal of placing Y-shaped endotracheal prostheses for the palliative treatment of central airway stenosis.

**Table 5:** Review of the literature.

Author/Year	Number of patients	Indication, *n* (%)	Stent Type	Duration, min (Mean ± SD)	Success of Deployment, *n* (%)	Mortality, *n* (%)	Relief of Symptoms, *n* (%)	Technical Complications, *n* (%)	Clinical Complications, *n* (%)
Dutau/2004 [23]	90	CAO 63(68.6) TEF 27(31.4)	Silicone	ND	85(94.4)	1(1.1)	84(93.3)	5(5.5)	1(1.1)
Han/2008 [24]	35	CAO 35(100)	Metallic	ND	31(88.6)	0	31(88.6)	4(11)	4(11.4)
Gompelmann/2013 [25]	43	CAO 43(100)	Metallic	ND	43(100)	1(2.3)	43(100)	0	1(2.3)
Monnier/2014 [16]	6	CAO 6(100)	Nitinol	111	6(100)	0	6(100)	0	0
Tsukioka/2015 [26]	12	CAO 12(100)	Silicone	ND	9(75)	1(8.3)	9(75)	0	3(25)
Madan/2016 [9]	38	CAO 30(78.9) TEF 4(10.5) Both 4 (10.5)	Metallic	38	37(97.4)	0	37(97.4)	1(2.6)	0
Sehgal/2017 [20]	27	CAO 21(77.8) TEF 6(29.6)	Silicone	48.9 ± 17.9	27(100)	1(3.7)	27(100)	3(11.1)	14(51.9)
Li/2020 [8]	10	CAO10(100)	Metallic	4 ± 1	10(100)	0	10(100)	0	0
Schulze/2022 [7]	27	CAO 17(62.9) TEF 10(37.1)	Nitinol	ND	21(68)	7(22.6)	ND	8(29.6)	0

CAO = Central airway obstruction; TEF = Tracheoesophageal fistula.

An important limitation of the present study is the relatively short follow-up period available for analysis. This constraint is intrinsically related to the retrospective multicenter design and to the clinical characteristics of the population. In many cases, stent placement was performed as an emergency procedure in severely dyspneic patients who required immediate airway stabilization and were subsequently discharged or transferred to other institutions to initiate or resume oncological treatment. As a result, long-term clinical data were not uniformly available across participating centers. This issue was further amplified by privacy regulations in some institutions, which prevented access to survival data.

The limitations of short follow-up in our cohort are consistent with those reported in the current literature. In a recent systematic review, Umar et al. [27] demonstrated that follow-up duration is inconsistently reported across studies, with several series providing no explicit long-term data. Moreover, the available evidence is highly heterogeneous, with substantial variability in survival outcomes, complication profiles, and functional improvement. Reported median survival after stent placement commonly falls between 3 and 6 months, reflecting the advanced disease stage of patients with malignant central airway obstruction and highlighting that long-term survival is determined primarily by the underlying malignancy rather than the stenting procedure itself.

A recent large retrospective study by Qi JC et al. [28] included 287 patients with malignant central airway obstruction. Patients treated with stents showed substantial improvement in airway stenosis. Functional outcomes including dyspnea, performance status, 6 min walk distance and health-related quality of life were significantly improved at 3- and 6-month follow-up compared with non-stent patients. Mean survival in the stent group was 5.1 months, indicating that while stent placement improves symptoms and short-term outcomes, survival remains limited and influenced by disease severity.

A retrospective series by Catarata et al. [29] included patients with advanced malignancies undergoing tracheobronchial stent placement. The study demonstrated that, in a total of 28 patients, 75% experienced immediate symptom relief after stenting, and performance status improved significantly post-procedure. Median survival was short (≈39 days), reinforcing that symptom relief is often the primary clinical benefit rather than prolonged survival.

In a large retrospective cohort of 102 patients receiving airway metallic stents for malignant central airway obstruction [5], the median survival after stenting was 4.1 months, with survival rates of 63.7% at 3 months and only 4.9% at 12 months. Notably, follow-up was nearly complete with only 4 out of 102 patients lost, but the rapid decline in survival emphasizes the aggressiveness of the underlying malignancies and the relative short duration of clinical observation in many published series. Moreover, multivariate analysis from this study highlighted that post-stenting radiotherapy and chemoradiotherapy were associated with better survival, underscoring the significance of combined interventional and oncologic treatment strategies in this setting.

These findings support the clinical rationale of focusing on immediate post-procedural outcomes and short-term follow-up, which represent the most consistent and meaningful endpoints in this scenario. Nevertheless, prospective studies with standardized long-term follow-up are warranted to better characterize disease progression, late stent-related complications, and survival trajectories.

## 5. Conclusions

The insertion of a metallic Y stent results in immediate palliation of dyspnoea in patients with malignant airway obstruction with low perioperative morbidity and mortality rates. Airway management, especially in terminal cancer patients and those with complex stenosis, requires a high level of expertise. The procedure is performed in highly experienced centers, where a multidisciplinary approach is necessary. As shown, patients can often access cancer treatments thanks to airway stabilization with recanalization and placement of a Y-shaped endotracheal tube.

### Limitations

This study has several limitations. First, it is a retrospective analysis conducted across multiple centers, each employing different techniques for airway stabilization. Additionally, the follow-up period was relatively short. Many patients were admitted to thoracic surgery or pulmonology departments just for the procedure and were then promptly discharged or transferred to other units. Furthermore, the prognosis for these patients is generally poor, as all had advanced-stage tumors.

## Figures and Tables

**Table 1:** List of centers involved, and number of patients included in the study.

Centers	Number of Patients (%)
Respiratory Diseases Department, University of Modena—Modena (Italy)	34(42.5)
Cardiothoracic Department, Freeman Hospital—Newcastle(UK)	15(18.8)
Thoracic Surgery Unit, University of Foggia—Foggia(Italy)	13(16.2)
Thoracic Sugery Unit, ‘Luigi Vanvitelli’ Hospital—Napoli(Italy)	9(11.5)
Thoracic Surgery Unit, University of Perugia—Perugia(Italy)	5(6.3)
Thoracic Surgery Unit, Papa Giovanni XXIII Hospital—Bergamo(Italy)	4(5.0)

**Table 2:** Patients’ and tumors’ characteristics.

Characteristic	All (*n* = 80)
Age—years (mean ± SD)	64.8 ± 9.6
Female sex—*n* (%)	45(56.2)
Clinical diagnosis—*n* (%)	
Lung tumors	66(82.5)
Esophagus tumors	9(11.2)
Other tumors	5(6.2)
Histology of lung tumors	
Squamous cell carcinoma	27(40.9)
Adenocarcinoma	21(31.8)
Other	10(15.1)
Microcitoma	8(12.2)
Histology of esophagus tumors	
Adenocarcinoma	5(55.6)
Squamous cell carcinoma	4(44.4)
Indication—*n* (%)	
Central airway obstruction	79(98.7)
Tracheoesophageal fistula	1(1.2)
Site of airway obstruction—*n* (%)	
Only carina	20(25.3)
Carina and main bronchus	22(27.8)
Only main bronchus	37(46.8)
Symptoms—*n* (%)	
Severe dyspnoea	80(100)
Stridor	10(12.5)
Chest pain	3(3.7)
Cough	2(2.5)

**Table 3:** Procedure’s details.

Outcome Data	All (*n*= 80)
Type of anaestesia, *n* (%)	
General	80(100)
Local	0
Type of bronchoscope, *n* (%)	
Rigid	80(100)
Flexible	0
Fluoroschopy, *n* (%)	15(18.7)
Type of stent, *n* (%)	
Metallic	75(93.7)
Silicone	5(6.2)
Recanalization maneuver before stent, *n* (%)	
Laser	25(31.2)
Balloon dilatation	45(56.2)
Duration of the procedure, min (mean ± SD)	36.7 ± 15.7
Successful placement, *n* (%)	76(95)
Stent dislocation	0

**Table 4:** Clinical outcomes.

Outcome Data	All (*n*= 80)
Periprocedural clinical complications, *n* (%)	
Arrhythmia	3(3.7)
Cardiac arrest	2(2.5)
Pneumothorax	2(2.5)
Patients who required ICU, *n* (%)	7(8.7)
Post procedure hospitalization, days (mean ± SD)	3.8 ± 3.1
Oncologic treatments after stent, *n* (%)	50(78.1)
In hospital mortality, *n* (%)	2(2.5)

## Data Availability

The data underlying this article are available in the article and its online.

## Abbreviations

The following abbreviations are used in this manuscript:

CAO	central airway obstruction
SEMS	self-expanded metallic stent
LOS	length of stay
CT	computed tomography
RMB	right main bronchus
LMB	left main bronchus
ND:YAG	neodymium-doped yttrium aluminum garnet
MBS	Modified Borg Scale
SD	standard deviation
